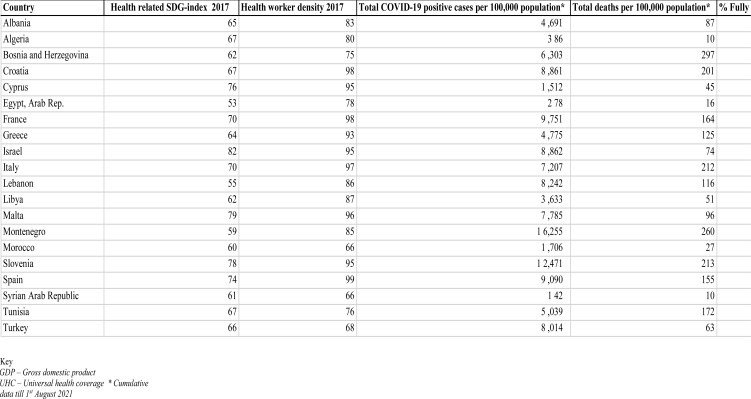# The impact of the COVID-19 pandemic on the Mediterranean region over 18 months: bridging the health outcomes and sustainable development goals

**DOI:** 10.4314/ahs.v22i4.61

**Published:** 2022-12

**Authors:** Sarah Cuschieri, Elizabeth Grech, Adrienne Gatt, Angelica Cutajar, Carine Vassallo, Daniel Zahra, Daniela Chatlani, Francesca Farrugia, Karen Cutajar, Leonie Mifsud, Maria Pia Tabone, Martina Azzopardi, Matthew Pace, Nicole Lorraine Mifsud, Nourah Aldousari, Raissa Baldacchino, Rebecca Caruana, Samuel Camilleri, Sarah Azzopardi, Sarah Cassar, Sarah Miceli Farrugia, Daniel Agius, Andrea Cuschieri

**Affiliations:** 1 Resident Academic, Department of Anatomy, Faculty of Medicine & Surgery, University of Malta, Msida, MSD2090 Malta; 2 Medical student, Faculty of Medicine & Surgery, University of Malta, Msida, MSD2090 Malta

**Keywords:** COVID-19, Mediterranean, Morbidity, Mortality, Vaccination, Sustainable Development Goals, Outcome Assessment

## Abstract

**Background:**

The COVID-19 pandemic spread across the globe, including across the Mediterranean basin. This region presents diversity in economy, culture, and societal affairs. We attempted to evaluate the impact of COVID-19 on the population and on the Sustainable Development Goals (SDGs), our aim being to aid in the development of COVID-19 national plans.

**Methods:**

Epidemiological data was obtained from ‘Our World in Data’ databases (January 2020 – July 2021). Case, mortality, and vaccination incidence comparisons were made across neighbouring countries. The SDG index, universal health coverage (UHC) and health workforce targets were collected for each country. Correlations between SDG targets and COVID-19 outcomes were analysed.

**Results:**

Similarities in morbidity and mortality outcomes were present across neighbouring countries, with a bidirectional relationship between cumulative fully vaccinated population and infectivity fatality rates. Positive relationships were present between SDG indexes, UHC and health workforces and COVID-19 cases, deaths, and vaccinations.

**Conclusion:**

At prima face, high-income countries seem to have sustained worse morbidity and mortality outcomes, despite having had better UHC and a greater health workforce in the pre-COVID-19 era however, one must also consider that factors such as health-seeking behaviour and underdiagnosis may have influenced this. Cross-border infectivity was, however, evident. Pan-Mediterranean action must therefore be taken to ensure COVID-19 transmissibility and mortality are reduced across borders, while ensuring an equitable health outcome across populations.

## Introduction

The Mediterranean basin has historically been the cradle of world civilization and has seen a wealth of historical empires emerge across the centuries 1,2. The Mediterranean Sea is situated between the European, Asian and African continents and is surrounded by various countries. This region shares a similar climate and has diverse cultural and social characteristics and was affected by the COVID-19 pandemic by early 2020 like the rest of the world. Considering the diversity in the economy, culture, and society across this region, it is anticipated that the COVID-19 impact will vary. Therefore, this study aims to address the research gap present regarding COVID-19 patterns in morbidity, mortality and vaccination across the Mediterranean region, while additionally attempting to address the impact on the attainment of the sustainable development goals in an attempt to aid in the development of COVID-19 national plans. The diversity in economy, culture and society present within the region was also considered. This article does not intend to identify causality for COVID-19 outcomes, nor does it attempt to evaluate pandemic governance across countries. As far as the authors know, this is the first study addressing the impact COVID-19 on the Mediterranean basin while considering the sustainable development goals.

## Methods

### Definitions

This was an observational study across the Mediterranean region. For the purposes of this study, the Mediterranean region was taken to consist of all the countries surrounding the Mediterranean Sea and independent islands within the Mediterranean Sea i.e., Spain, France, Italy, Slovenia, Croatia, Bosnia and Herzegovina, Montenegro, Albania, Greece, Turkey, Malta, Cyprus, Syria, Israel, Lebanon, Egypt, Libya, Tunisia, Algeria, and Morocco. For ease of comparison analyses, the Mediterranean countries were sub-categorised into three groups/regions: (i) the countries that form part of the OECD (Organisation for Economic Co-operation and Development), i.e., Spain, France, Italy, Slovenia, Croatia, Greece, Turkey, and Israel, (ii) the remaining countries that form part of the European continent (Albania, Bosnia and Herzegovina, Cyprus, Malta, Montenegro) and (iii) the Middle East/North Africa (MENA) region (Egypt, Lebanon, Libya, Morocco, Syria, Tunisia).

The Sustainable Development Goals (SDGs) have been set by the United Nations General Assembly to act as a guideline for countries to attain a better future for all. There are seventeen different SDGs that cover various aspects of life. The SDG index is an assessment of each country's overall performance in terms of the SDGs, giving equal weight to each goal 3. The index for 2019 (which represents the pre-COVID-19 era) and the index for 2021 (which represents the COVID-19 era) were obtained for all the Mediterranean countries and compared.

COVID-19 affected various SDGs but had a substantial impact on the third SDG: “Good Health and Well-being”. Two of the targets for this SDG were considered in our study: “Universal Health Coverage” (UHC) and “Health worker density” targets.

UHC considers the “*coverage of essential health services (defined as the average coverage of essential services based on tracer interventions that include reproductive, maternal, newborn and child health, infectious diseases, non-communicable diseases and service capacity and access, among the general and the most disadvantaged population)”* 3. This target was considered appropriate for this study since COVID-19 is an infectious disease that affects all aspects of health. Health worker density measures the availability of healthcare workers, a factor known to have been a challenge since the onset of COVID-19. This was therefore considered an appropriate target to consider for this study.

### Data sources

All data collected covered the period between the onset of COVID-19 in Mediterranean countries and the 1st August 2021 (i.e., week 30 of 2021). Epidemiological data (COVID-19 cases, deaths and vaccination) was obtained from the ‘Our World in Data’ (OWID) database 4.

The SDG index for each Mediterranean country was obtained from the 2021 Sustainable Development report 3, while health worker density for the year 2017 (last measured) for each country was obtained from the Global Burden of Disease Study 2019 5.

### Data analyses

The weekly epidemiological data obtained from the OWID database was combined to fit within a fiscal month distribution. The monthly cases and deaths were then converted to incidence rates per 100,000 population for ease of comparison. A heatmap was created using Microsoft Excel®, to illustrate the degree of COVID-19 transmission per month, where green represented mild, yellow represented moderate and orange-red represented high transmission.

Each Mediterranean country was analysed in relation to the surrounding neighbouring countries, i.e., countries that are geographically adjacent to each other, or which are landlocked using Microsoft Excel®.

Total vaccinated population data for each country was converted into cumulative vaccination per 100,000 population and comparisons were made between countries through a heatmap for the months of January, March, May, and July 2021. The heatmaps were created using the freely available software ‘Map Chart’ (https://mapchart.net/europe.html). The cumulative fully vaccinated population per 100,000 population was also compared to infectivity fatality rates (IFR) of the corresponding month. The IFR was calculated using Microsoft Excel®, by dividing the number of deaths by the number of confirmed infective cases for each month, multiplied by 100. Correlation analyses were performed using the IBM SPSS Statistics for Mac version 21 (IBM Corp., Armonk, N.Y., USA), between the SDG index, UHC, health worker density, cumulative COVID-19 cases (18 months), COVID-19 deaths (18 months) and total proportion of vaccinated population (up until 1st August 2021), in order to establish whether there were any relationships between SDG factors and the COVID-19 situation.

A summary of the study's methodological pathway that was undertaken is shown in Supplement Figure 1.

**Supplement figure 1:**
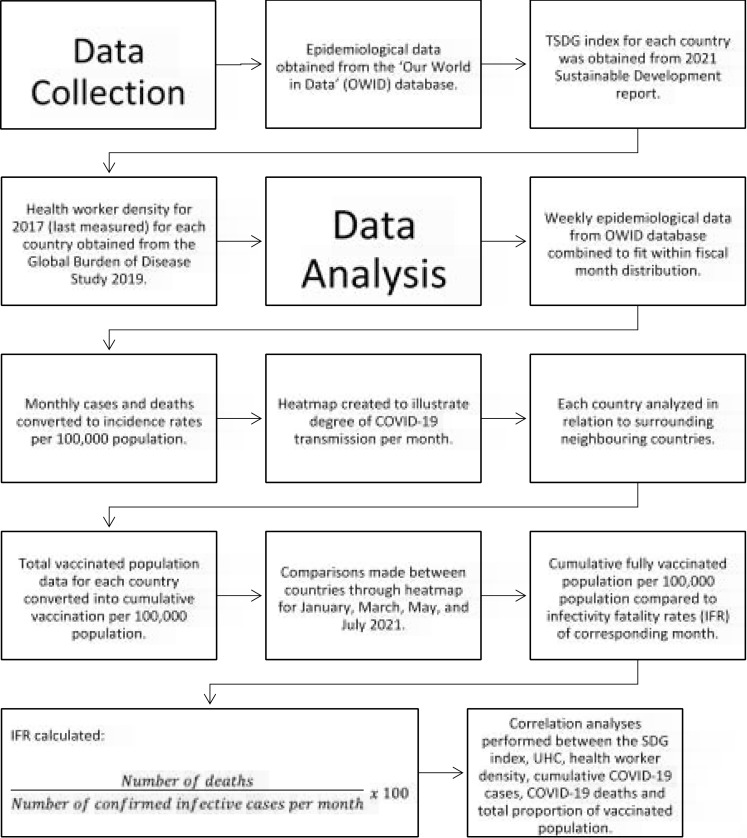
Flow chart illustrating method used to establish whether there were any relationships between SDG factors and the COVID-19 situation.

## Results

The total number of COVID-19 cases reported across the Mediterranean region over a period of 18 months (February 2020 – July 2021), was 26,857,728, with 473,932 deaths. The Syrian Arab Republic reported the lowest number of COVID-19 cases (142 per 100,000 population) while Montenegro reported the highest number of COVID-19 cases (16,255 per 100,000 population) across these 18 months, as shown in the nest graph: Figure 1A. The lowest number of COVID-19 related deaths were reported by both Algeria and the Syrian Arab Republic (10 per 100,000 population in both cases), while Bosnia and Herzegovina reported the highest number of deaths (297 per 100,000 population), as shown in the nest graph: Figure 1B.

**Figure 1A:**
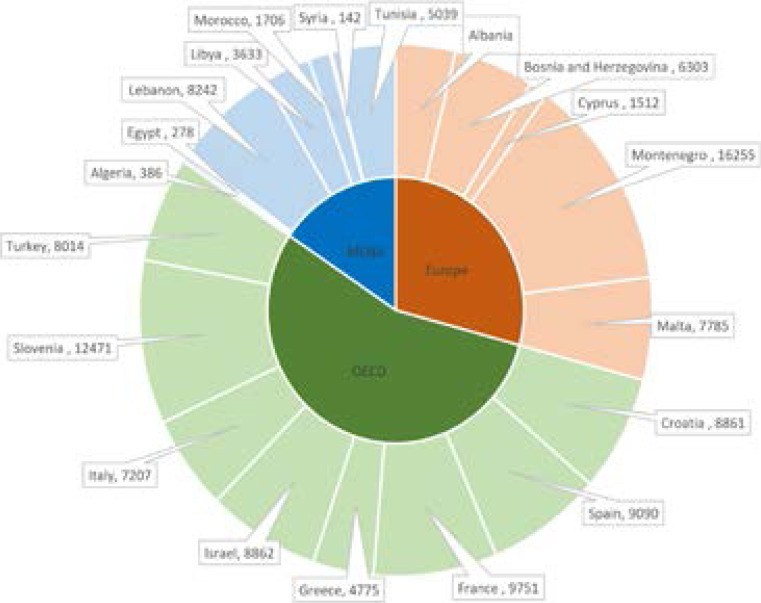
Nest graph comparing the total COVID-19 cases per 100,000 population of Mediterranean countries according to MENA, OECD and Europe regions

**Figure 1B:**
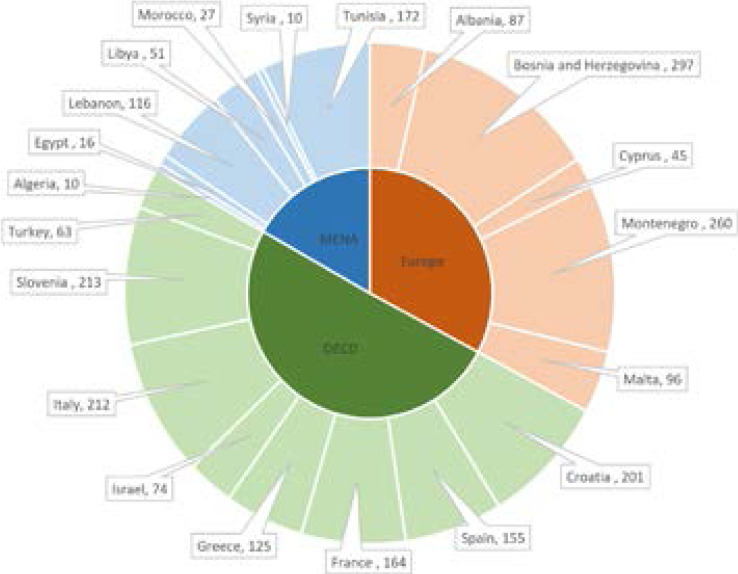
Nest graph comparing the total COVID-19 deaths per 100,000 population of Mediterranean countries according to MENA, OECD and Europe regions

**Figure 2:**
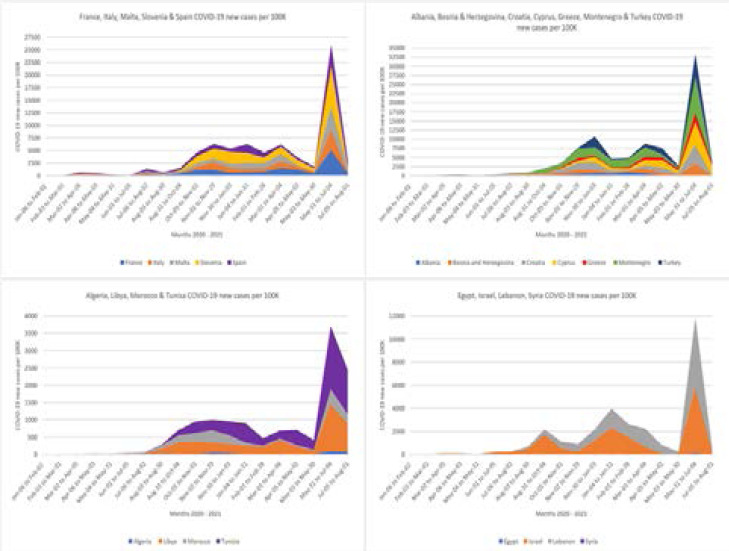
Vaccination comparisons per 100,000 population between January and August 2021 across Mediterranean countries

Croatia, France, Greece, Israel, Italy, Lebanon, and Spain reported the first COVID-19 cases in February 2020, followed by the remaining Mediterranean countries as shown in Table 1. COVID-19 transmission varied across time throughout the Mediterranean region, with relatively low cases numbers between February and July 2020 as opposed to the succeeding months, with some exceptions. Table 1 is a heat map which illustrates the changes in infectivity across countries and time for all the Mediterranean countries. Most of the Mediterranean countries were observed to have the highest infectivity rate between August 2020 and May 2021, with some exceptions (Table 1). Interestingly, neighbouring countries showed similar infectivity trends which is suggestive of cross-border infectivity as shown in Supplement Figure 2.

**Table 1:** Heatmap showing the reported monthly new COVID-19 cases per 100,000 population across all Mediterranean countries between February 2020 and August 1^st^ 2021. (Green represents low COVID-19, Yellow medium COVID-19, Orange-Red high COVID-19 transmission)

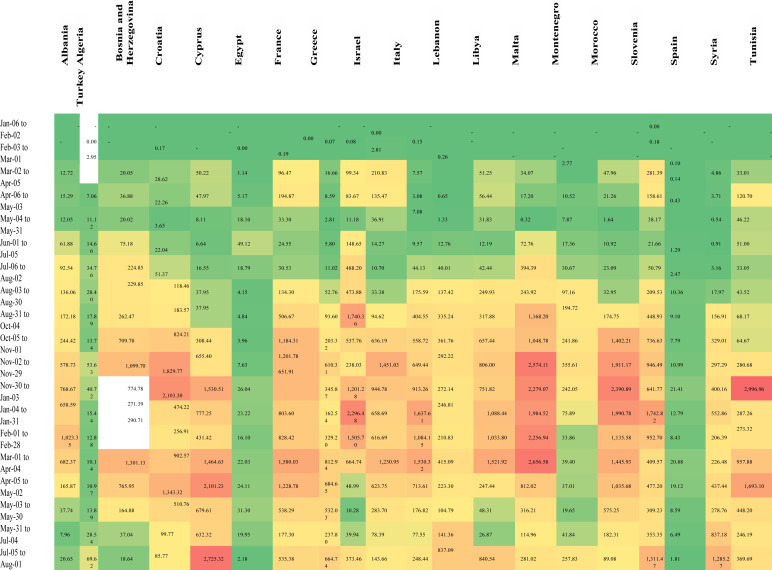

**Supplement Figure 2:**
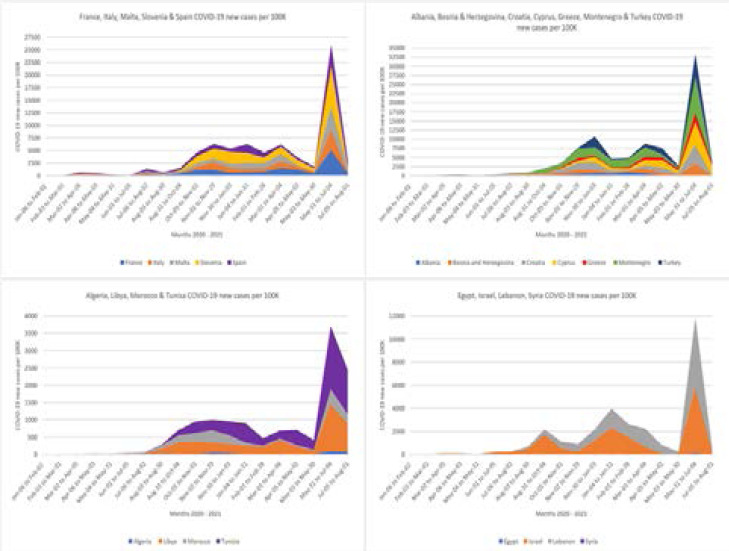
New COVID-19 cases per 100,000 population: comparisons between Mediterranean region and neighbouring countries

**Figure 3:**
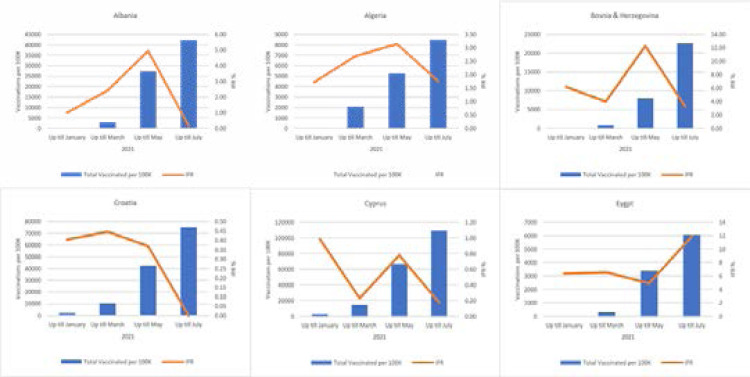

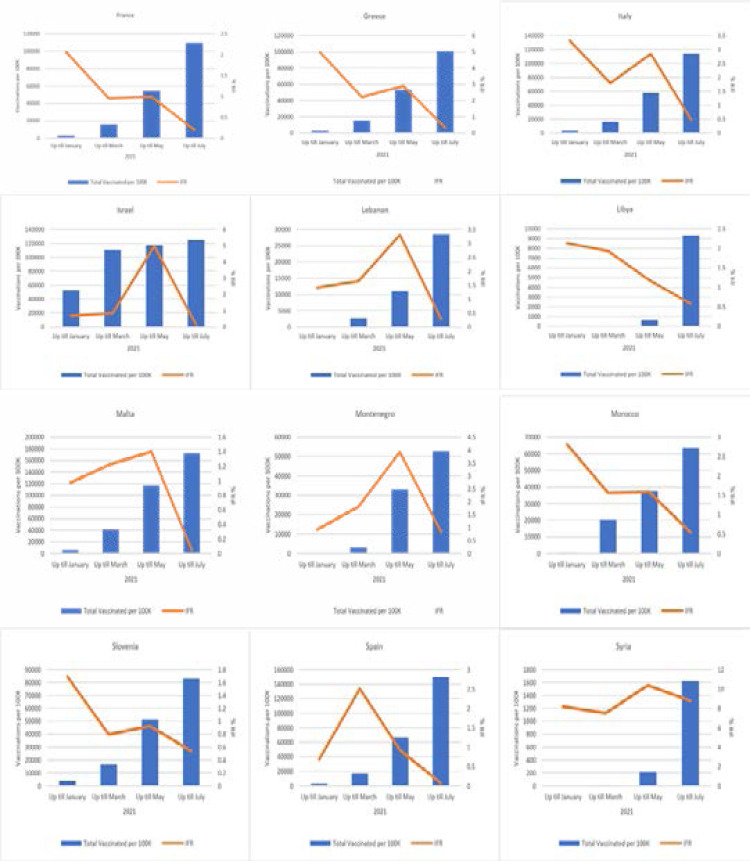

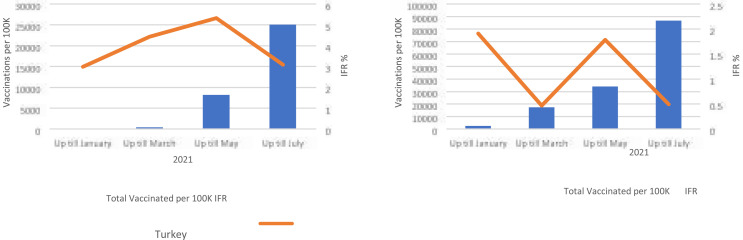
Comparison analyses between total vaccinations per 100,000 population and infectivity fatality rate (IFR) across each Mediterranean country

COVID-19 vaccination rollouts within the Mediterranean Region began at different time periods, with countries that form part of the European Union, along with Israel, having had access to vaccines at an earlier stage^6^,^7^. Indeed, a progressive vaccination roll out could be seen across the Mediterranean Region, as seen in Supplement Figure 3, with Israel and Malta having the fastest vaccination roll out, while the Syrian Arab Republic and Egypt appeared to have the slowest roll out. Despite this, a bidirectional relationship between total vaccination per 100,000 population and the infectivity fatality rate (IFR) is evident across all countries. In fact, as a substantial proportion of each Mediterranean country's population was vaccinated, the IFR started to decline, as shown in Figure 2. Indeed, a sharp decline in IFR was observed from May 2021 onwards for most of the countries. This was especially pronounced for Albania, Bosnia & Herzegovina, Croatia, Israel, Lebanon, Malta, Montenegro, and Turkey.

## COVID-19 and sustainable development goals (SDGs)

COVID-19 had an impact on many different sectors namely the economy, the environment, and the social dimension, resulting in setbacks in terms of SDGs. Regardless, on comparing the 2019 SDG index (pre-COVID-19) to the 2021 SDG index (COVID-19 era) as reported by the United Nations, most of the Mediterranean countries, except for Algeria, Malta, and the Syrian Arab Republic, were noted to have experienced an increase in the SDG index, as shown in Table 2. However, a positive correlation was established between the 2021 SDG-index and total COVID-19 cases (R: 0.51 p=0.02) and deaths (R: 0.52 p=0.02) per 100,00 population. Similarly, universal health coverage (UHC) for 2019 was positively correlated with total COVID-19 cases (R: 0.58 p=0.01) and deaths (R: 0.52 p=0.02) per 100,00 population, as well as with the proportion of the population fully vaccinated (R:0.82 p=<0.01) within the Mediterranean Region. Indeed, the Mediterranean countries of Algeria, Egypt and Morocco had the lowest UHC pre-COVID-19 and have reported low COVID-19 infectivity, mortality, and vaccination rates, as shown in Table 2. Positive correlations were also noted between “Health worker density” (2017), total COVID-19 cases (R: 0.56 p=0.01) and the proportion of the population fully vaccinated (R:0.72 p=<0.01). Additionally, a positive correlation was present between “Health Related SDG” (2017) and the proportion of the population fully vaccinated (R:0.75 p=<0.01).

**Table 2:** Comparisons between the Gross domestic product (GDP), various sustainable development goal factors, total COVID-19 cases, deaths per 100,000 population and the proportion of the population fully vaccinated in each Mediterranean country

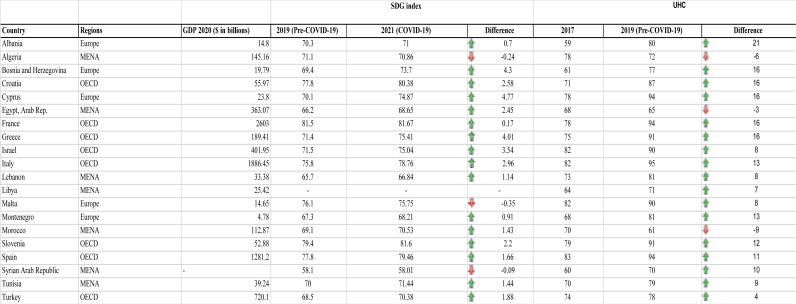

## Discussion

COVID-19 transmission across the Mediterranean region A number of Mediterranean countries, including Malta, Greece, Algeria and Morocco reported their first cases to have been individuals that travelled back from either Italy or France 8–10. Countries in the East Mediterranean (Lebanon and Israel) and Libya reported their first COVID-19 case to have arrived from countries outside Europe 11–13. COVID-19 has challenged every population across the world undeterred by the country's income, characteristics, culture, or current affairs. The Mediterranean European countries of Italy and France appeared to have played a major role in the initial transmission of COVID-19 cases to the rest of the Mediterranean region. a number of Mediterranean countries, including Malta, Greece, Algeria and Morocco reported their first cases to have been individuals that travelled back from either Italy or France 8–10. Countries in the East Mediterranean (Lebanon and Israel) and Libya reported their first COVID-19 case to have arrived from countries outside Europe 11–13.

Countries that followed rigid containment measures saw a decline in cases, as reported in Israel and Malta 9,17. The initial phase (first wave) of COVID-19 saw most Mediterranean countries implementing lockdowns and travel restrictions including border closure with an associated decrease in morbidity and mortality rates 14–17. Swift relaxation of measures, however, resulted in a steady increase in cases and deaths 17,18 Cross-border transmissibility and mortality were observed across the 18 months under study. The initial phase (first wave) of COVID-19 saw most Mediterranean countries implementing lockdowns and travel restrictions including border closure with an associated decrease in morbidity and mortality rates 14–17. Although the knee-jerk reaction of countries has been to restrict geographical borders and act as an isolated nation, it became evident that cross-border and international joint actions are required to tackle this pandemic effectively 19. Especially since, as observed in this study, cross-border transmission was clearly visible despite the aforementioned restrictions.

## Challenges of COVID-19

COVID-19 challenged many country's healthcare systems, especially when fragmented systems were present to begin with as in Egypt, Italy and Lebanon 20–22. Other countries, such as Malta, showed resilience due to good COVID-19 healthcare system preparatory actions 23. Some countries within the East and Southern Mediterranean Region such as Lebanon and Libya experienced additional challenges as COVID-19 occurred on a background of economic, political, and armed conflicts 21,24. Such societal problems might have hindered population access to COVID-19 testing and healthcare, as well as disrupted public health surveillance and test-trace-isolate approaches. This could also have potentially resulted in inaccurate data due to lack of ability to report the full extent of the COVID-19 situation. Indeed, it was reported that the infectivity rate reported in Libya is debatable due to inadequate public health infrastructure 25. These factors could explain the low infectivity and mortality rates noted in this study, especially among the MENA countries. Another potential factor contributing to this is the lack of COVID-19 testing capacities, as noted by Algeria and Egypt 22,26.

## Impact of COVID-19 on SDGs

Positive correlations were established in this study between universal health coverage and SDG index with COVID-19 cases and deaths. The Mediterranean region consists of countries that range from high-income to low-income, providing the ideal platform to attempt to assess the effect of the economy on COVID-19 outcomes. The COVID-19 pandemic has challenged healthcare systems as well as the livelihoods of populations, with high-income countries having reported suffering higher morbidity and mortality27. This was indeed evident in this study and coincides with the positive correlations established between universal health coverage and SDG index with COVID-19 cases and deaths. High income countries tend to be more economically stable with the ability to provide a better healthcare service to the population. It is therefore not surprising that a positive correlation was evident between UHC and the proportion of the population fully vaccinated. Furthermore, it is expected that a higher income country would have a higher health worker density. A positive correlation was also established between health worker density and the proportion of the population fully vaccinated. It is, in fact, expected that a higher income country would have a higher health worker density. The greater the workforce and the more economically stable, the more equipped a country is to plan and execute a fast vaccination rollout, as seen in Israel and in Malta. This is however, provided that a low vaccine hesitancy among the population exists 28,29. Although vaccination strategies and availability varied across the Mediterranean Region, as the vaccination rollout started to progress, it was evident that the goal of the vaccine to reduce mortality came into effect. This was seen through a decrease in IFR as the proportion of the vaccinated population increased.

It has been noted that COVID-19 has setback the attainment of the SDGs 3. However, the SDG 2021 index was noted to show an incline in most countries from the pre-COVID SDG index 2019, with some exceptions. This observation may be a result of underestimations or inaccurate data due to the disruptions in data reporting and time lags in statistics due to the pandemic 3.

Other limitations that need to be acknowledged include the fact that the study is based on freely available online COVID-19 data. The accuracy of the data is dependent on the source and the data reporting. The low COVID-19 cases reported in some countries could be the result of issues in reporting and contact tracing, while deaths might have been missed, especially those that occurred in private residences or in remote places. Data on hospital admissions and intensive care unit admissions was lacking for most countries which prevented us from evaluating the impact of COVID-19 on the various healthcare systems. These limitations might have affected the epidemiological interpretation and may have led to some inaccuracies. Every effort was made by the contributors to identify accurate data originating from reliable sources; however, it does not exclude the possibility of some missing data.

## Conclusion

COVID-19 transmission initially seemed inexorable and affected all countries irrelevant of their economic, social, cultural, and current affairs. At prima face, high-income countries seem to have sustained worse morbidity and mortality outcomes, despite having had better UHC and a greater health workforce in the pre-COVID-19 era, and despite exhibiting faster vaccination rollouts with positive population results. However, one must also consider that factors such as varying health-seeking behaviour and underdiagnosis may have influenced this. Pan-Mediterranean action must therefore be taken to ensure that COVID-19 transmissibility and mortality are reduced across borders, as has already been called for by European countries experts 30,31. One of the key findings of this study was that cross-border infectivity appeared to have played a major role in COVID transmission. In addition, although the pandemic did indeed setback the attainment of the SGDs, most Mediterranean countries still exhibited an increase in SGD index. Furthermore, rigid restriction measures were proven to be effective in that decrease in morbidity and mortality rates was evident in countries which employed these measures. Moreover, higher vaccination rates were associated with decreased IFR. These findings strengthen the argument that pan-Mediterranean action must be taken to allow countries within the area to reduce COVID-19 transmissibility and mortality across borders. This is especially true for neighbouring countries and must be done while exhibiting solidarity to ensure equal access to vaccination and healthcare across the region with an equitable health outcome across populations. It is up to the governments of the various countries within the area to create a dialogue between themselves and to come up with and implement strategies that will benefit the various countries and regions within the Mediterranean.

## Figures and Tables

**Table:**